# Supplementary material for: Colonic mucosal associated invariant T cells in Crohn’s disease have a diverse and non-public T cell receptor beta chain repertoire
Source: PLoS One. 2023 Nov 3;18(11):e0285918. doi: 10.1371/journal.pone.0285918 (PMC10624325; doi:10.1371/journal.pone.0285918)
Supplement: S1 Checklist — (DOCX) [file pone.0285918.s001.docx]

STROBE Statement—checklist of items that should be included in reports of observational studies

|  | Item No. | Recommendation | Page  No. | Relevant text from manuscript |
| --- | --- | --- | --- | --- |
| **Title and abstract** | 1 | (*a*) Indicate the study’s design with a commonly used term in the title or the abstract | n/a | n/a |
|  |  | (*b*) Provide in the abstract an informative and balanced summary of what was done and what was found | 2 | The transcriptome of MAIT cells sorted from blood and intestinal lamina propria cells from colectomy recipients were compared with other CD8^+^ T cells. Colon biopsies from an additional ten CD patients and ten healthy controls (HC) were analyzed by flow cytometry. TCR genes were sequenced from individual MAIT cells from these biopsies and compared with those of MAIT cells from autologous blood. |
| Introduction | | | |  |
| Background/rationale | 2 | Explain the scientific background and rationale for the investigation being reported | 3-5 | Too large to reproduce here |
| Objectives | 3 | State specific objectives, including any prespecified hypotheses | 5 | We describe a more detailed analysis of MAIT cells in CD than has previously been published, including both flow cytometry and full genome transcriptome profiling of these cells in the intestinal mucosa. Furthermore, we describe paired TCR alpha and beta chain gene sequences in individual MAIT cells from colon biopsies, paired with TCR beta sequences from autologous blood MAIT cells, to report on the clonality, diversity, and potentially public nature of these semi-invariant cells in the GI tract in health and disease. |
| Methods | | | |  |
| Study design | 4 | Present key elements of study design early in the paper | 6-10 | Too large to reproduce here |
| Setting | 5 | Describe the setting, locations, and relevant dates, including periods of recruitment, exposure, follow-up, and data collection | 6 | … surgery from 12/23/09-11/18/13 at Virginia Mason Medical Center in Seattle, WA, USA… colonoscopic biopsies …from 8/6/12-10/24/16 at Virginia Mason Medical Center in Seattle, WA, USA |
| Participants | 6 | (*a*) *Cohort study*—Give the eligibility criteria, and the sources and methods of selection of participants. Describe methods of follow-up  *Case-control study*—Give the eligibility criteria, and the sources and methods of case ascertainment and control selection. Give the rationale for the choice of cases and controls  *Cross-sectional study*—Give the eligibility criteria, and the sources and methods of selection of participants | 6 | … six CD patients, six UC patients, and six patients without IBD as the indication for surgery… colonoscopic biopsies were obtained from ten healthy screening colonoscopy recipients and ten CD patients |
|  |  | (*b*) *Cohort study*—For matched studies, give matching criteria and number of exposed and unexposed  *Case-control study*—For matched studies, give matching criteria and the number of controls per case | n/a | n/a |
| Variables | 7 | Clearly define all outcomes, exposures, predictors, potential confounders, and effect modifiers. Give diagnostic criteria, if applicable | n/a | n/a |
| Data sources/ measurement | 8* | For each variable of interest, give sources of data and details of methods of assessment (measurement). Describe comparability of assessment methods if there is more than one group | 6-10 | Too large to reproduce here |
| Bias | 9 | Describe any efforts to address potential sources of bias | n/a | n/a |
| Study size | 10 | Explain how the study size was arrived at | n/a | n/a |

Continued on next page

| Quantitative variables | 11 | Explain how quantitative variables were handled in the analyses. If applicable, describe which groupings were chosen and why | 6-10 | Too large to reproduce here |
| --- | --- | --- | --- | --- |
| Statistical methods | 12 | (*a*) Describe all statistical methods, including those used to control for confounding | 9-10 | Comparisons between groups of flow cytometry data and other continuous variables did not presume a Gaussian distribution. Therefore, analysis of variance used a Kruskal-Wallis test for unpaired data and a Friedman test for paired comparisons. Two-way comparisons were performed using a Mann-Whitney *U*-test for unpaired comparisons and a Wilcoxon signed-rank test where data points could be paired (e.g. were from the same donor). Specific tests employed are specified in figures and/or their legends. Given the exploratory nature of these analyses, *P*-values were not adjusted for multiple comparisons. |
|  |  | (*b*) Describe any methods used to examine subgroups and interactions | n/a | n/a |
|  |  | (*c*) Explain how missing data were addressed | n/a | n/a |
|  |  | (*d*) *Cohort study*—If applicable, explain how loss to follow-up was addressed  *Case-control study*—If applicable, explain how matching of cases and controls was addressed  *Cross-sectional study*—If applicable, describe analytical methods taking account of sampling strategy | n/a | n/a |
|  |  | (*e*) Describe any sensitivity analyses | n/a | n/a |
| Results | | | | |
| Participants | 13* | (a) Report numbers of individuals at each stage of study—eg numbers potentially eligible, examined for eligibility, confirmed eligible, included in the study, completing follow-up, and analysed | 6 | … surgical resections of six CD patients, six UC patients, and six patients without IBD… colonoscopic biopsies were obtained from ten healthy screening colonoscopy recipients and ten CD patients |
|  |  | (b) Give reasons for non-participation at each stage | n/a | n/a |
|  |  | (c) Consider use of a flow diagram | n/a | n/a |
| Descriptive data | 14* | (a) Give characteristics of study participants (eg demographic, clinical, social) and information on exposures and potential confounders | 6 | Clinical and demographic details about these donors are presented in Supplementary table 1. |
|  |  | (b) Indicate number of participants with missing data for each variable of interest | n/a | n/a |
|  |  | (c) *Cohort study*—Summarise follow-up time (eg, average and total amount) | n/a | n/a |
| Outcome data | 15* | *Cohort study*—Report numbers of outcome events or summary measures over time | n/a | n/a |
|  |  | *Case-control study—*Report numbers in each exposure category, or summary measures of exposure | n/a | n/a |
|  |  | *Cross-sectional study—*Report numbers of outcome events or summary measures | n/a | n/a |
| Main results | 16 | (*a*) Give unadjusted estimates and, if applicable, confounder-adjusted estimates and their precision (eg, 95% confidence interval). Make clear which confounders were adjusted for and why they were included | n/a | n/a |
|  |  | (*b*) Report category boundaries when continuous variables were categorized | n/a | n/a |
|  |  | (*c*) If relevant, consider translating estimates of relative risk into absolute risk for a meaningful time period | n/a | n/a |

Continued on next page

| Other analyses | 17 | Report other analyses done—eg analyses of subgroups and interactions, and sensitivity analyses | n/a | n/a |
| --- | --- | --- | --- | --- |
| Discussion | | | | |
| Key results | 18 | Summarise key results with reference to study objectives | 19 | We therefore compared gene expression in CD4^-^ MAIT cells to that of other CD8^+^ T cells in the intestines, as well as the blood, and compared blood and intestinal CD4^-^ MAIT surface protein immunophenotypes and TCR sequences in CD patients and HC. |
| Limitations | 19 | Discuss limitations of the study, taking into account sources of potential bias or imprecision. Discuss both direction and magnitude of any potential bias | n/a | n/a |
| Interpretation | 20 | Give a cautious overall interpretation of results considering objectives, limitations, multiplicity of analyses, results from similar studies, and other relevant evidence | 19-23 | Too large to reproduce here |
| Generalisability | 21 | Discuss the generalisability (external validity) of the study results | 19-23 | Too large to reproduce here |
| Other information | |  | | |
| Funding | 22 | Give the source of funding and the role of the funders for the present study and, if applicable, for the original study on which the present article is based | 24 | We thank the Wilske Pioneer Fund, and internal award at Virginia Mason Medical Center, for funding this research. |

*Give information separately for cases and controls in case-control studies and, if applicable, for exposed and unexposed groups in cohort and cross-sectional studies.

**Note:** An Explanation and Elaboration article discusses each checklist item and gives methodological background and published examples of transparent reporting. The STROBE checklist is best used in conjunction with this article (freely available on the Web sites of PLoS Medicine at http://www.plosmedicine.org/, Annals of Internal Medicine at http://www.annals.org/, and Epidemiology at http://www.epidem.com/). Information on the STROBE Initiative is available at www.strobe-statement.org.
