# Supplementary figures and images for: Colonic mucosal associated invariant T cells in Crohn’s disease have a diverse and non-public T cell receptor beta chain repertoire
Source: PLoS One. 2023 Nov 3;18(11):e0285918. doi: 10.1371/journal.pone.0285918 (PMC10624325; doi:10.1371/journal.pone.0285918)

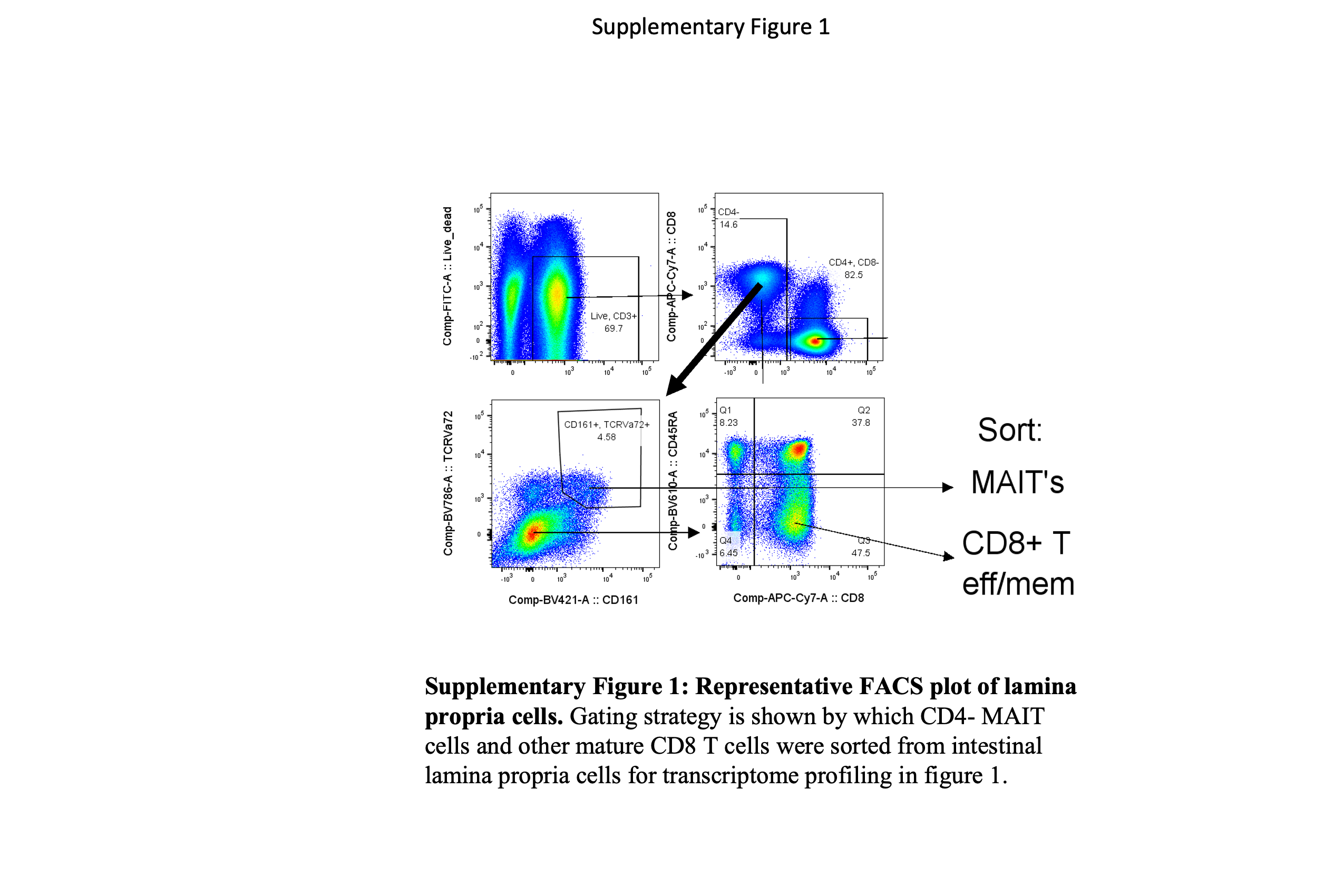

Supplement: S1 Fig — Gating strategy is shown by which CD4- MAIT cells and other mature CD8 T cells were sorted from intestinal lamina propria cells for transcriptome profiling in Fig 1. (TIFF) [file pone.0285918.s002.tiff]

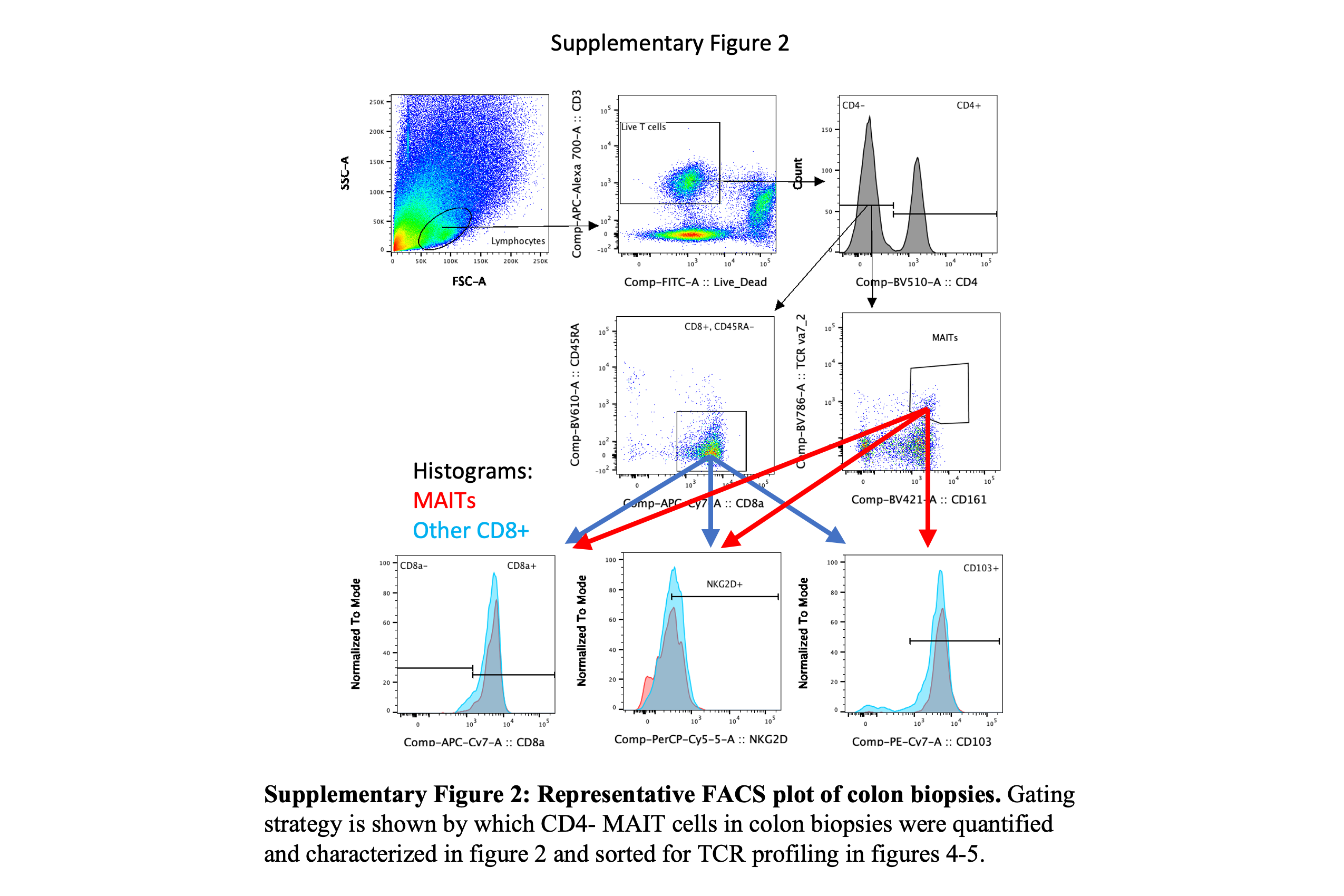

Supplement: S2 Fig — Gating strategy is shown by which CD4- MAIT cells in colon biopsies were quantified and characterized in Fig 2 and sorted for TCR profiling in Figs 4 and 5. (TIFF) [file pone.0285918.s003.tiff]

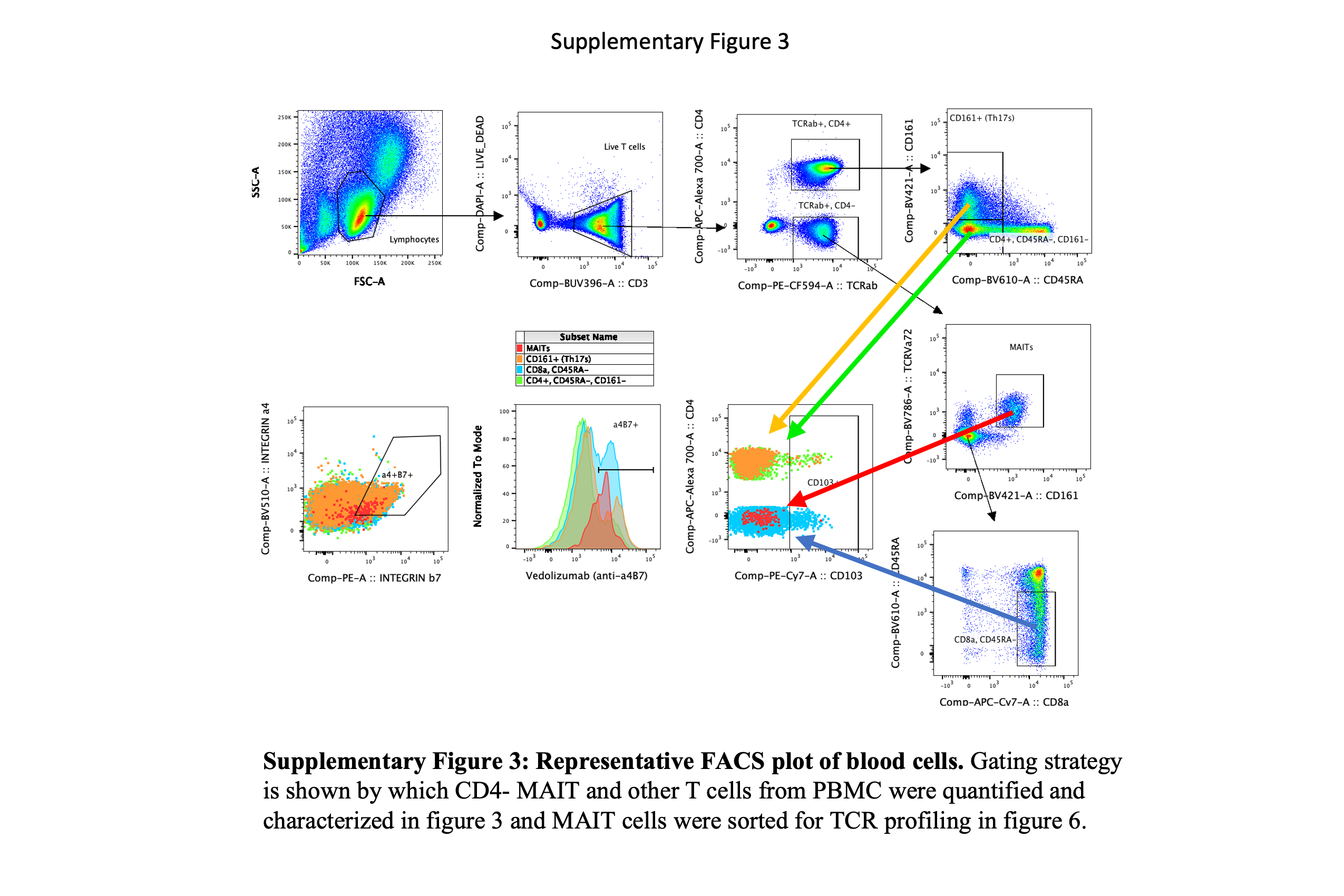

Supplement: S3 Fig — Gating strategy is shown by which CD4- MAIT and other T cells from PBMC were quantified and characterized in Fig 3 and MAIT cells were sorted for TCR profiling in Fig 6. (TIFF) [file pone.0285918.s004.tiff]
